# Epidemiology, antibiotic consumption and molecular characterisation of *Staphylococcus aureus* infections – data from the Polish Neonatology Surveillance Network, 2009–2012

**DOI:** 10.1186/s12879-015-0890-3

**Published:** 2015-04-01

**Authors:** Dorota Romaniszyn, Anna Różańska, Jadwiga Wójkowska-Mach, Agnieszka Chmielarczyk, Monika Pobiega, Paweł Adamski, Ewa Helwich, Ryszard Lauterbach, Maria Borszewska-Kornacka, Ewa Gulczyńska, Agnieszka Kordek, Małgorzata Bulanda

**Affiliations:** Chair of Microbiology, Jagiellonian University Medical College, 18 Czysta Street, Kracow, 31-121 Poland; Institute of Nature Conservation, Polish Academy of Sciences, Kraków, Poland; Clinic of Neonatology and Intensive Neonatal Care, Institute of Mother and Child, Warsaw, Poland; Clinic of Neonatology, Jagiellonian University Medical College, Kraków, Poland; Clinic of Neonatology and Intensive Neonatal Care, Warsaw Medical University, Warsaw, Poland; Clinic of Neonatology, Polish Mother’s Memorial Hospital-Research Institute, Lodz, Poland; Department of Neonatal Diseases, Pomeranian Medical University, Szczecin, Poland

## Abstract

**Background:**

Our aim was to determine and characterize S. aureus (SA) isolated from infections in newborns for antibiotic resistance, virulence factors, genotypes, epidemiology and antibiotic consumption.

**Methods:**

Prospective surveillance of infections was conducted. Data about antibiotic treatment were analyzed. Antimicrobial susceptibility was assessed. PCR amplification was used to detect resistance and virulence genes. Typing methods such as PFGE, spa-typing and SCCmec were used.

**Results:**

SA was found to be associated with 6.5% of infections. Methicillin-Resistant *Staphylococcus aureus* accounted for 32.8% of SA-infections. An incidence of MRSA-infections was 1.1/1000 newborns. MRSA-infections were diagnosed significantly earlier than MSSA-infections in these newborns (14th day vs. 23rd day (p = 0.0194)). MRSA-infections increased the risk of newborn’s death. Antibiotic consumption in both group was similar, but a high level of glycopeptides-usage for MSSA infections was observed.

In the MRSA group, more strains were resistant to erythromycin, clindamycin, gentamicin and amikacin than in the MSSA group. *Hla* gene was present in 93.9% of strains, and *seg* and *sei* in 65.3% of strains, respectively. One dominant clone was found among the 14 MRSA isolates. Fifteen strains belonging to SCC*mec* type IV were spa-t015 and one strain belonging to SCC*mec* type V was spa-t011.

**Conclusions:**

Results obtained in the study point at specific epidemiological situation in Polish NICU (more detailed studies are recommended).

High usage of glycopeptides in the MSSA infections treatment indicates the necessity of antimicrobial stewardship improvement and introducing molecular screening for early identification of infections.

**Electronic supplementary material:**

The online version of this article (doi:10.1186/s12879-015-0890-3) contains supplementary material, which is available to authorized users.

## Background

Systemic infections caused by *Staphylococcus aureus* (SA) are still common among newborns in neonatal intensive care units (NICUs) [1]. The danger of *S. aureus* is attributed to its resistance to many antibiotics and high virulence [2]. To our knowledge, there have been no studies on the epidemiology of SA in patients of NICUs in Poland. In fact, there have been no systemic surveillance of such infections in this vulnerable population.

The primary aim of this study was to determine and characterize *S. aureus* isolates from newborns according to resistance, virulence factors, genotypes and epidemiology. Secondly, we assessed the level of antibiotic usage and possible risk factors for SA infection (separately for Methicillin-sensitive SA (MSSA) and Methicillin-resistant SA (MRSA)). These factors included: clinical (use of devices: central/peripheral venous catheter – CVC/PVC, mechanical ventilation – MV, continuous positive airway pressure – CPAP, or total parenteral nutrition – TPN, length of stay in hospital until infection, and breast/trophic feeding) and demographic data.

## Methods

Utilization of data collected in the Polish Neonatology Surveillance Network (PNSN) for scientific purposes was approved by the Bioethics Committee of Jagiellonian University Medical College – no. KBET/227/B/2012. All data entered into the electronic database and analyzed during this study were previously anonymised and de-identified. The PNSN is a prospective national surveillance system for the most relevant infections in VLBW infants (with birth weight < 1500 g) in Poland. Details of the following variables were collected for all VLBW newborns: birth weight and gestational age, gender, multiple pregnacy, type of delivery and information of the situation in time of delivery, for example chorioamnionitis, general status of newborns by Apgar score: at 1 and 5 minutes and Critical Risk Index for Babies, CRIB) and others. Participation in PNSN is voluntary and confidential, the surveillance covered over 20% of VLBW infants born in the regions where the NICUs were located.

### Study population

Prospective surveillance of infections was conducted between 2009–2012 at five tertiary academic NICUs that lead the perinatal care, in hospitals (designed by letters A, B, C, E, F) that participated in the PNSN, and included 1768 newborns. The labor of 135 newborns was complicated by chorioamnionitis, while 429 infants were delivered after premature rupture of membranes (PROM). All episodes of infection were subjected to recording, regardless of the time of occurrence of the first symptoms. Case patients were defined according to Gastmeier *et al.* [3] with modification, as very low birth weight (VLBW) neonates who demonstrated clinical signs and/or symptoms of bloodstream infection (BSI) or pneumonia.

Early-onset infection was defined as infection diagnosed within 3 days after delivery. Device-associated infection was defined as infection diagnosed in newborns who had a device (central/peripheral venous catheter or mechanical ventilation or continuous positive airway pressure) placed within the 48-hour period before infection onset. Device utilization ratio (calculated by dividing the number of days with devices by the total number of patient/days, pds) was: 0.41 for CVC, 0.21 for PVC, 0.38 for MV, 0.28 for CPAP and 0.47 for TPN.

SA-infection was defined as culture-proven infection with isolation of SA. MSSA (or MRSA) infection was defined as culture-proven infection with isolation of MSSA (or MRSA).

### Bacterial isolates

Collecting and identifying bacterial strains were performed in the local microbiology laboratories. Various diagnostic specimens were collected for culture and assessment of the microbial aetiology of infections. Altogether, 58 SA strains were isolated – and were considered to be the aetiological factor of infection. In this group, 49 strains were used for further analysis: most were from cases of pneumonia (20) and from BSI (19); nine were not stored. Strains in local laboratory were stored in −20°C, in laboratory of Chair of Microbiology were stored in −70°C. Isolates were identified by the automated identification system (VITEK 2, bioMerieux, Poland).

### Susceptibility testing

All strains were tested using disk diffusion antimicrobial susceptibility methods according to current guidelines of the European Committee on Antimicrobial Susceptibility Testing (Clinical breakpoints tables v. 3.1; http://www.eucast.org v.3.1). E-tests for vancomycin and teicoplanin (bioMérieux, Paris, France) were also performed for all the isolates.

The MRSA resistance phenotype was detected using a cefoxitin disc (30 μg) according to the EUCAST guidelines. The macrolide-lincosamide (MLS) resistance phenotype of the isolates was determined according to a previously published protocol [4].

### DNA isolation

DNA was extracted from isolates using the Genomic Mini kit (A&A Biotechnology, Gdynia, Poland) according to the manufacturer’s instructions.

### Polymerase chain reaction (PCR) screening for resistance genes

PCR amplification was used to detect the *mec*A gene using previously published primers [5]. As controls, *S. aureus* ATCC 33591 (*mec*A+) and *S. aureus* ATCC 25923 (*mec*A−) were employed. Erythromycin resistance genes (*erm*A, *erm*B, *erm*C, and *msr*) were detected by multiplex PCR, and amplification of a 456 bp fragment of the *mup*A gene (mupirocin resistance gene) was performed by single PCR [6,7].

### Antibiotic treatment

Data about antibiotic treatment were entered into the database by the ward personnel based on the physicians’ orders. Precise information about the type of drug, daily dose, and length of treatment for each antibiotic was collected and used for the calculation of two indicators: duration of treatment and defined daily dose in reference to each case of infection.

The aggregate sum of the number of days during which at least one dose of antibiotic was received for each antibiotic used (days of treatment, DOTs) was expressed in days and the defined daily dose (DDD), according to the ATC/DDD system of the World Health Organization (Anatomical Therapeutic Chemical, group “J01”). Only antibiotics for systemic use were taken into account.

Antibiotic usage for treatment (until cure) was assessed for 56 cases; two cases were excluded from analysis due to death of the infant within the first seven days of illness (unsuccessful treatment) and lack of data on treatment of one newborn.

### Virulence factor screening

*S. aureus* isolates were checked for the presence of selected virulence genes: *tsst* (toxic shock syndrome toxin), *sea, seb, sec, seg, seh, sei, sej* (staphylococcal enterotoxins A, B, C, G, H, I, J), *eta, etb* (exfoliative toxins A and B), *luk*E (LukDE leucocidin), *pvl* (Panton-Valentine leucocidin, and *hla* (staphylococcal alpha haemolysin) using PCR with previously published primers [8-10]. The following *S. aureus* strains were used as positive controls: 2535/07 (*eta*+, *etb*+, *seg*+, *sei*+), 8977/99 (*sea*+, *sec*+, *seg*+, *sei*+), 6616/09 (*seb*+, *tsst*+, *pvl*+), 2027/06 (*sea*+, *seh*+), and 1034/05 (*sea*+, *seg*+, *sei*+, *sej*+). The strains used as controls were kindly provided by Prof. Marek Gniadkowski, National Medicines Institute, Warsaw, Poland.

### Pulsed-Field Gel Electrophoresis (PFGE)

Analysis of the genetic similarity between SA isolates was performed by PFGE method in accordance with a previously published protocol [11]. Restriction enzyme digestion was performed with 25 U of *Sma*I enzyme in Tango buffer (ThermoScientific, USA). Electrophoresis was conducted in a CHEFIII PFGE unit applying the parameters: block 1- starting pulse 5 s, ending pulse 12 s, voltage 6 V/cm, run time 11 h, block 2- starting pulse 20s, ending pulse 60 s, voltage 6 V/cm, run time 13 h. The GelCompar (Applied Maths, Sint-Martens-Latem, Belgium) was used for cluster analysis using the Dice coefficient and unweighted pair group method with arithmetic mean. Isolates that clustered ≥ 95% were considered as an epidemic clone. *S. aureus* strain ATCC 11632 was used as reference.

### Spa sequencing

spa typing was performed as described previously [12], using the spa typing website (http://www.spaserver.ridom.de/) that was developed by Ridom GmbH (Münster, Germany).

### SCC*mec* typing

Staphylococcal cassette chromosome *mec* (SCC*mec*) typing was performed as described previously [13]. The following *S. aureus* strains were used as positive controls: ATCC-BAA 1762 (SCC*mec* IV), ATCC-BAA 2094 (SCC*mec* V), and ATCC-BAA 1681 SCC*mec* II).

### Statistical methods

The statistical analysis was based on two main groups of techniques. Comparison of the ratio of MSSA/MRSA was performed with a contingency test supplemented by odds ratio analysis. To determine the relationship between continuous and categorical variables, Student’s *T* test and ANOVA were used. If the distribution of the continuous variable did not fit a normal distribution, the analysis was conducted with nonparametric equivalents of parametric tests: Wilcoxon test for Student’s *T* test, and Kruskal-Wallis test for ANOVA. The relationship between two continuous variables was analyzed with Pearson Regression or its non-parametric alternatives. Because of the categorical nature of the effect and combined – numeric as well as categorical – types of predictors, the model was constructed for binominal distribution of dependent variables and the logit-linked function. P-values of <0.05 were considered to be statistically significant.

## Results

The total number of SA infection was 58 cases, these the incidence of SA-infections was 3.3% and 1.0/1,000 pds. The incidence of MRSA infections (19 cases) was 1.1%. No significant association was found between time of hospitalization in NICU and the risk of MRSA. The number of MRSA infections in the group of SA-infections – proportion was 32.8 (range from 8.3% to 42.1% in different centers). The most common SA-infections were BSI (55.2%) and pneumonia (39.7%). MRSA was observed more often among BSI, statistical significance was not observed but a strong trend was noted MRSA (p = 0.0515; odds ratio, OR 2.2807, 95% Confidence Interval, CI 0.7198–7.2265). MRSA was not associated with a central/peripheral line catheter (p = 0.8085, OR 0.963, 95% CI 0.2952–3.1417).

All SA-infections were diagnosed on average at day 21 of stay, (median: 17th day). MRSA-infections were diagnosed significantly earlier than MSSA-infections, on average 14th day vs. 23rd day; there was no relationship between the use of devices and prevalence of SA-infections (either MSSA or MRSA).

The fatality case rate was higher among newborns with MRSA infection than with MSSA infection (10.5% vs. 0.0%). The gestational age, birth weight, and other factors of newborns with MRSA was similar to those with MSSA (Table 1).

**Table 1:** **Characteristics of newborns with SA-infections**

	**Infants with MSSA-infections [N = 39]**	**Infants with MRSA-infections [N = 19]**	**P-value**
**Risk factors of patients**	**average (95%CI)**	**average (95%CI)**	
Birth weight [grams]	873.7 (791.0–956.5)	948.3 (817.3–1079.4)	0.304
Gestational age [weeks]	27.7 (26.8–28.6)	27.2 (25.6–28.8)	0.316
CRIB	5.5 (3.5–7.5)	2.4 (1.9–6.8)	0.049
Apgar (1 min.)	4.9 (4.3–5.6)	4,7 (3.7–5.8)	0.860
Apgar (5 min.)	6.2 (5.6–6.8)	6.0 (4.8–7.2)	0.817
length of stay in hospital [days]	56.2 (49.1–63.2)	48.4 (37.1–59.8)	0.197
length of hospital stay until infection [days]	23.8 (19.7–28.5)	14.7 (9.8–19.7)	0.019
DOT	11.2 (9.6–12.7)	12.3 (7.7–16.8)	0.885
DDD	30.2 (22.6–37.8)	26.0 (16.4–33.5)	0.457
**Risk factors of patients**	**number (%)**	**number (%)**	**OR (95%CI) P-value**
Female gender	19 (48.7)	7 (36.8)	0.614 (0.200–1.889) 0.393
Breast feeding	4 (10.5)	1 (5.6)	0.486 (0.051–4.677) 0.542
Trophic feeding	21 (55.3)	12 (66.7)	1.470 (0.477–4.525) 0.418
Total parenteral nutrition	25 (65.8)	15 (79.0)	2.1 (0.583–7.571) 0.306
Fatal cases	0 (0.0)	2 (10.5)	n/a; n/a; 0.039

CRIB, clinical risk index for babies; DOT, the aggregate sum of the number of days during which at least 1 dose of antibiotic was received for each one received, days of treatment; DDD, defined daily dose expressed in days.

Multivariate analysis also showed no association between the ward studied, phenotype of the isolate of MSSA/MRSA, type of infection, birth weight, and duration of pregnancy.

### Antibiotic treatment

The average antibiotic consumption was similar between MSSA and MRSA cases. The DOT average was 11.2 for MSSA cases and 12.3 for MRSA cases; the DDDs were equal between the groups (30.2 and 26.0, respectively, Table 1). However, statistically significant differences were observed in DOT between the studied wards (ward B was significantly shorter than ward E, p = 0.002) and in DDD (ward F had lower consumption than ward A, p = 0.0049). There were no significant differences between DDD and length of therapy of SA-infections depending on phenotype (MRSA vs. MSSA).

Antibiotics used most often for treatment of SA infections (both methicillin-susceptible and –resistant; Table 2) were glycopeptides, beta-lactams and aminoglycosides. For beta-lactams DOT values were significantly higher for MSSA than for MRSA (Z = −2.77787, p = 0.0052; MRSA mean 3.5; SD 1.37; median 3.5, max 5; for MSSA mean 7.8; SD 4.15; median 7; max 21).

**Table 2:** **Distribution of individual antibiotic groups usage in MRSA (18 cases) and MSSA (28 cases) infections**

**Antibiotic groups**	**DOT [%]**		**OR (95%CI) P-value**	**DDD [%]**		**OR (95%CI) P-value**
	**MRSA**	**MSSA**		**MRSA**	**MSSA**	
Aminoglycosides	17.2	14.9	0.976 (0.697–1.367) 0.967	31.7	28.0	1.366 (1.096–1.704) 0.561
Beta-lactams	6.3	31.7	0.146 (0.092–0.233) 0.005	19.0	40.9	0.338 (0.264–0.435) 0.879
Fluoroquinolones	6.0	0.0	n/a	9.3	0.0	n/a
Glycopeptides	63.6	43.0	2.296 (1.766–2.985) 0.427	33.4	24.9	1.513 (1.208–1.895) 0.533
Lincozamides	2.7	1.1	2.562 (1.008–6.512) 0.221	0.8	1.0	0.775 (0.250–2.408) 0.073
Macrolides	4.2	5.8	0.723 (0.393–1.330) 0.832	2.8	4.4	0.629 (0.348–1.138) 0.072
Trimethoprim/sulfamethoxazole	0.0	3.0	n/a	0.0	0.2	n/a
Metronidazole	0.0	0.5	n/a	0.0	0.6	n/a
**Total**	**100.0**	**100.0**		**100.0**	**100.0**	

OR, odds ratio; 95%CI, 95% Confidence Interval; DOT, the aggregate sum of the number of days during which at least 1 dose of antibiotic was received for each one received, days of treatment; DDD, defined daily dose expressed in days; n/a not applicable.

Diagram showing antibiotics consumption in the study population is presented in Additional file 1.

### Resistance and virulence

The MRSA phenotype was found for 16 isolates (32.6%) and the inducible MLS (iMLS) phenotype was observed in 11 isolates (22.4%). Seven strains exhibited both phenotypes. Four isolates (8.2%) with M phenotype (resistant to erythromycin but susceptible to clindamycin) were found. All strains were susceptible to ofloxacin, vancomycin and teicoplanin. The MIC_50_ for vancomycin was 1.5 mg/ml and for teicoplanin was 1.0 mg/ml.

Molecular characterisation showed that all MRSA isolates carried the *mec* gene. Only three MLS strains had *erm* genes (*erm*A was found in two isolates and *erm*B in one isolate). One strain had the *mup* gene.

*S. aureus* isolates were tested for the presence of genes encoding for virulence factors (VFs). The differences in distributions of the VFs were analysed. Detailed data are presented in Table 3: no association was found between number of VFs and resistance to numbers of antimicrobial classes.

**Table 3:** **Virulence factors among isolates and resistance to antimicrobials**

**Virulence factor**	**All [N = 49]; (n, %)**	**MRSA [N = 16]; (n, %)**	**MSSA [N = 33]; (n, %)**
***hla***	46 (93.9)	16 (100)	30 (90.9)
***sea***	8 (16.3)	1(6.3)	7 (21.2)
***seb***	0	0	0
***sec***	7 (14.3)	4 (25)	3 (9.1)
***seg***	32 (65.3)	15 (93.8)	17 (51.5)
***seh***	1 (2.0)	1 (6.3)	0
***sei***	32 (65.3)	15 (93.8)	17 (51.5)
***sej***	4 (8.2)	0	4 (12.1)
***eta***	7 (14.3)	4 (25)	3 (9.1)
***etb***	0	0	0
***tsst***	9 (18.4)	0	9 (27.3)
***lukE***	19 (38.8)	0	19 (57.6)
***pvl***	0	0	0
**Resistance**	**All [N = 49]; (%)**	**MRSA [N = 16]; (%)**	**MSSA [N = 33]; (%)**
**E**	16 (32.6)	10 (62.5)	6 (18.2)
**DA**	12 (24.5)	7 (43.7)	5 (15.1)
**OFX**	0	0	0
**GN**	3 (6.1)	2 (12.5)	1 (3.0)
**TOB**	6 (12.2)	2 (12.5)	4 (12.1)
**AK**	6 (12.2)	3 (18.7)	3 (9.1)
**VA**	0	0	0
**TEC**	0	0	0

Legend: E-erythromycin, DA-clindamycin, OFX – ofloxacin, GN – gentamycin, TOB – tobramycin, AK – amikacin, VA – vancomycin, TEC – teicoplanin.

*hla* (staphylococcal alpha haemolysin), *sea, seb, sec, seg, seh, sei, sej* (staphylococcal enterotoxins A, B, C, G, H, I, J), *eta, etb* (exfoliative toxins A and B), *tsst* (toxic shock syndrome toxin), *luk*E (LukDE leucocidin), *pvl* (Panton-Valentine leucocidin).

### Typing

**Figure 1:**
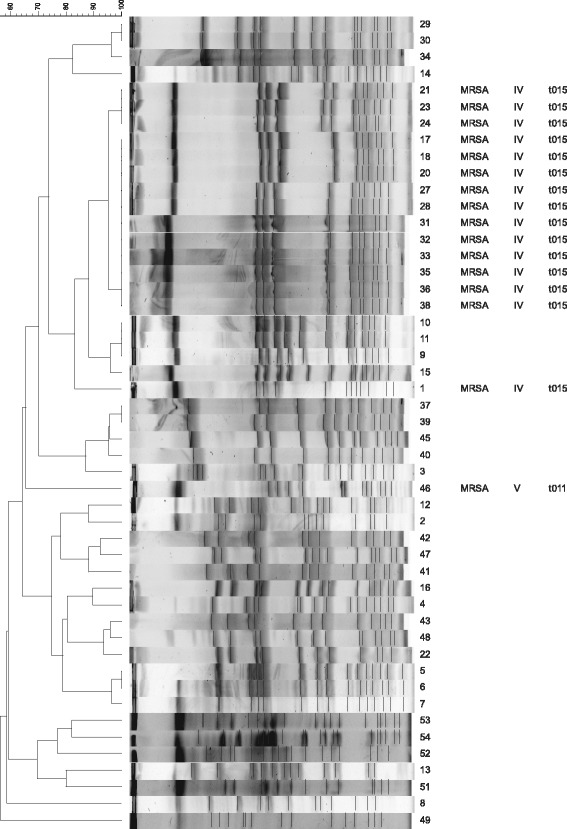
**Results of PFGE, SCC** ***mec*** **and** ***spa*** **typing of** ***S.aureus*** **isolates.**

MSSA isolates showed very different pulsotypes; dominant clones were not detected. Cluster analysis based on PFGE of the 33 isolates showed 23 pulsotypes, some of which were less than 70% similar, suggesting a genotypically variable population. Isolates with identical pulsotypes were usually derived from different patients in the same NICU during the same period of time. Analysis of *Sma*I macro-restriction profiles of the 16 MRSA isolates revealed one dominant clone (14 isolates). The two remaining MRSA isolates had different PFGE patterns (Figure 1). MRSA isolates belonging to the dominant clone were derived from one NICU designed by the letter F. Two spa types and two SCC*mec* were observed: spa-t015 in the case of 15 isolates belonging to the SCC*mec* type IV and spa-t011 in one isolate belonging to the SCC*mec* type V.

## Discussion

*Staphylococcus aureus* is still one of the most important causes of infections in NICU patients, and usually represents a large part of the aetiology of infection. For example, in the UK, Vergnano reported a prevalence of about 18% [14], which was similar to the level in the U.S. [15], whereas in Germany the prevalence was 10.2% [16]. In the group of neonates in the present study, SA-infections were observed 2–3 times less frequently. Similar results have been obtained in the field of drug resistance, especially MRSA, in late- and early-onset infections. A number of multicentre studies, however, found a higher proportion of MRSA. In Japan, MRSA strains accounted for more than 88% [17] of SA infections, and in Cyprus, 68% [18], of SA infections.

In contrast, in the US (only in VLBW newborns) the prevalence of MRSA infection was 0.4% [19], while in Poland and the UK, it was 1.1% [14]. Furthermore, according to Vergnano’s data, the proportion of infections with the MRSA phenotype in late infections was a little over 10% of SA-infections, which was much less than in the group examined in the present study [14].

The difference in findings between studies may be due to the fact that the United Kingdom was one of the first countries in the world to introduce wide mandatory surveillance of MRSA [20,21]. For example, beginning in 2010, screening for MRSA in most patients admitted for elective hospital was instituted [22]; this action underscores the fact that MRSA colonization was considered an important risk factor for this aetiology of infection [23]. Implementation of the UK guidelines resulted in a reduction of MRSA infections in health care settings in general [24]. Furthermore, this reduction has had a direct impact on the prevalence of MRSA in the general population; thus, the risk of colonization of pregnant women, young parents and NICU staff also has dropped especially since MRSA is not only found in hospitals, but often occurs as “community-acquired” infections [25,26].

The fact that there was no relationship between MRSA infections resulting from invasive procedures and late-onset MRSA infections should be discussed with special attention. A number of authors pointed to an increasing risk of infections with multidrug-resistant (MDR) strains during hospitalisation and resulting from invasive procedures. Lessa *et al.* showed that MRSA infections among newborns observed most often were late-onset: median age at disease onset was 28 days [19]. These results suggest that infection with MDR strains should be also considered at the early stage of hospitalisation. No relationship between MRSA infections resulting from invasive procedures could be made with the huge number of invasive procedures among this group of patients. Hocevar *et al.* showed that MRSA constituted about 33% of device-associated infections, which is consistent with the results of this study [27].

Rational antimicrobial treatment is an important element of surveillance for drug-resistant strains [28]. The results of this study reveal the methodological difficulties associated with the assessment of the consumption of antibiotics by neonates. In similar studies, other authors used indicators such as DOT, LOT (the number of days where at least one dose of any antibiotic was received), DDD or PDD (prescribed daily dose) [29,30]. In the present study, there were no significant differences in DOT or DDD as determined by analysis of the average consumption of all groups of antibiotics used in the treatment of SA infections. Stratification of antibiotic consumption according to individual groups showed, however, that the indicator used, (DOT/DDD), has an impact on significance of the differences in therapeutic regimens used for MRSA and MSSA. In case of beta-lactams the level of antibiotic consumption was statistically significantly higher only in case of assessment with DOT usage. Therefore, analysis of antibiotic consumption should not be based on only one parameter, and the data should be stratified in order to be able to determine meaningful differences [31].

The data about antibiotic consumption show high usage of glycopeptides in both groups: MRSA-infections and MSSA-infections, when expressed as DOT. Rapid diagnostic molecular methods (which enable rapid assessment of the need to implement or discontinue therapy with vancomycin) should be implemented to decrease glycopeptide consumption, because glycopeptides are not easy to use in neonates [32,33]. On the other hand, the study of Brzychczy-Włoch *et al.* showed a high percentage of Gram-positive *mec*A-positive infections in NICUs [34]. For this reason vancomycin should not be eliminated from use among NICUs but effort should be made toward lowering the vancomycin consumption.

As expected, the haemolysin gene was present in almost all studied isolates with occasional exceptions. In contrast, genes encoding exfoliative toxins were rare: no such patients were among our study population. The low frequency of *eta* and *etb* coincides with the result of the other studies [2,35].

In recent years, MRSA producing the Panton-Valentine leucocidin toxin have emerged as increasingly common causes of community-acquired infections [36,37]; however, we did not find *pvl*-positive isolates, which was similar to other studies [24].

Of enterotoxin genes detected, those within the *egc* (enterotoxin gene cluster) *seg + sei* were the most frequent (65.3%), which also was shown by Rasmussen [35]. These enterotoxin genes were found frequently in isolates from patients but their presence did not mean the production of toxin was high enough to cause food poisoning. These superantigens may enhance the persistence of infection but their role as a virulence factors is still purely speculative [38].

Among leucocidins, the prevalence of *lukE* was relatively high and associated with MSSA but not MRSA strains. Moreover, the *tsst* gene was not detected in any of the MRSA isolates, which was similar to other studies [2].

There have been some studies showing a link between virulence and resistance [39,40], no such association was found in this study.

A number of molecular methods have been developed and implemented for typing MRSA isolates including SCC*mec*, PFGE, and spa typing [2]. Most of the MRSA strains (and only MRSA) isolated in this study belonged to the epidemic clone: these strains had identical pulsotypes, belonged to the same SCC*mec* type and spa type, and came from one of the surveyed centers.

Reports from NICUs showed that it is not easy to determine the incidence of MRSA-infections. However, results from the present study, combining detailed epidemiological analyses with molecular studies of SA, allowed a much broader evaluation of these infections: development, course and severity of the infection were compared with resistance and virulence of the strain, and the type of populations where they were observed.

Several potential limitations should be considered in the interpretation of data presented here. First, the number of isolates investigated was low. Another important limitation was the lack of a control group – surveillance was conducted only on a group of very-low birth weight infants.

## Conclusions

The key points of results of our study are:

- the MRSA infections were diagnosed significantly earlier than MSSA-infections (14 vs. 23 day of hospitalization)
- low proportion of MRSA infections in the group of SA-infections (32.8%) which additionally were less frequent than reported in other studies pointing at specific epidemiological situation in Polish NICU (more detailed studies are recommended)
- high usage of glycopeptides in the MSSA infections treatment which indicates the necessity of antimicrobial stewardship improvement and introducing molecular screening for early identification of infections

## Additional file

Additional file 1:
**Antibiotics consumption in the study population.**
