# Supplementary figures and images for: Epidemiology, antibiotic consumption and molecular characterisation of Staphylococcus aureus infections – data from the Polish Neonatology Surveillance Network, 2009–2012
Source: BMC Infect Dis. 2015 Apr 1;15:169. doi: 10.1186/s12879-015-0890-3 (PMC4389670; doi:10.1186/s12879-015-0890-3)

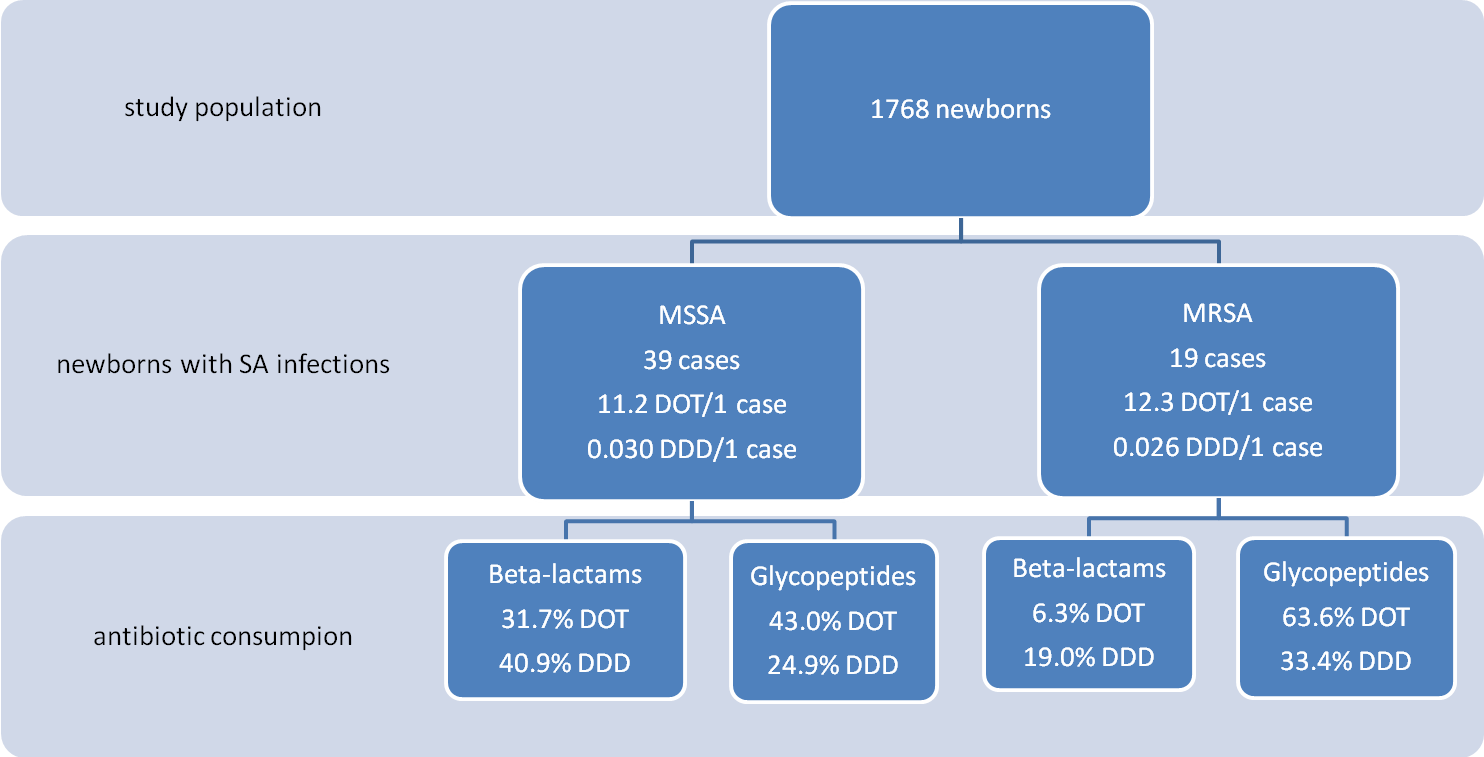

Supplement: Additional file 1: — Antibiotics consumption in the study population. [file 12879_2015_890_MOESM1_ESM.docx]
